# Bionomics of malaria vectors in Lao PDR, 2018–2020: entomological surveillance as a key tool for malaria elimination

**DOI:** 10.1186/s12936-023-04754-5

**Published:** 2023-10-21

**Authors:** Sébastien Marcombe, Santi Maithaviphet, Rita Reyburn, Khamfong Kunlaya, Khambang Silavong, Bouasy Hongvanthong, Viengxay Vanisaveth, Viengphone Sengsavath, Vilasack Banouvong, Keobouphaphone Chindavongsa, Boualam Khamlome, Matthew Shortus

**Affiliations:** 1 VCC-SEA, Vector Control Consulting, Vientiane, Lao PDR; 2 Center for Malariology, Parasitology and Entomology, Ministry of Health, Vientiane, Lao PDR; 3 World Health Organization, Saphanthong Tai Rd, Vientiane, Laos

**Keywords:** Laos, Malaria, Anopheles, Vector, Bionomics, Entomology surveillance

## Abstract

**Background:**

The Lao PDR National Strategic Plan for malaria control and elimination for year 2021–2025 emphasizes the importance of routine entomological surveillance being conducted in areas with high transmission and in active malaria foci in elimination targeted areas. The collection of entomological surveillance data that is closely linked to recent epidemiological data is crucial for improving impact, as it contributes to the evidence package that supports operational and strategic decision-making of national malaria programmes, as they accelerate their last mile of elimination.

**Methods:**

The Center for Malariology Parasitology and Epidemiology (CMPE) entomology team conducted entomological surveillance activities at 13 sentinel sites in 8 provinces and at active transmission foci sites from 2018 to 2020. The techniques used for the mosquito collection were indoor and outdoor human landing collections (from houses and from cultivation areas) and cattle baited net trap collections.

**Results:**

There were 5601 *Anopheles* mosquito females captured and identified throughout the study, on both human and cow bait. They represented 15 different species or species complexes. The primary malaria vectors as well as the secondary vectors were present in all collection sites in the south, indicating that people living in these rural areas with high malaria incidence are exposed to the vectors. The vectors were highly zoophilic, but they still bite humans throughout the night with a high peak of activity before midnight, both indoors and outdoors. Overall, 17% of the malaria vectors were collected indoors when the people are sleeping. This confirms the importance of bed net use during the night. Thirty-two percent of primary and secondary vectors were collected outdoors at times when people are usually awake and outdoors, which shows that people are exposed to potentially infectious mosquitoes and the importance of personal protection at these times. The findings showed that residual transmission may occur outdoors in the villages, and outside the villages in cultivation fields and forested areas. Epidemiological data showed that transmission was higher in surveillance sites which were targeted as part of a malaria response rather than sentinel sites.

**Conclusions:**

Understanding where and how transmission is persisting, monitoring and mapping vector species distribution in areas with active transmission, monitoring biting trends, and designing evidence based and effective vector control interventions are critical to accelerating progress toward malaria elimination. In this context, the role of entomological surveillance combined with epidemiological data should be considered as a cornerstone in achieving malaria elimination.

**Supplementary Information:**

The online version contains supplementary material available at 10.1186/s12936-023-04754-5.

## Background

In Laos, malaria parasite transmission occurs mostly in remote and forested areas particularly in the southern part of the country where 3552 cases were reported in 2020 (MOH national health information system) [1]. Since 1992, the country has implemented a nationwide malaria control programme based on (i) vector control, with the use of long-lasting insecticidal nets (LLINs), and (ii) treatment with rapid diagnostic test and early response with artemisinin-based combination therapy (ACT). There has been a large decrease of malaria burden over the last decade in the country (40% reduction of case incidence between 2015 and 2020) [2] and following these encouraging results, the Lao Ministry of Health (MoH) has planned to eliminate the disease by 2030 [3]. The current National Strategic Plan for Malaria Control and Elimination (2021–2025) is the second part (phase II) of a three phases approach to eliminate all forms of malaria. This includes strengthened interventions targeted to the southern part of the country to reduce the malaria burden, while also expanding and enhancing efforts to eliminate malaria and prevent reintroduction in low burden focal areas across the whole country. Specifically, the phase II goal of the NSP is to eliminate *Plasmodium falciparum* malaria in the entire country by the end of 2023 and to eliminate all species of malaria parasite (*Plasmodium vivax* and *P. falciparum*) along with the entire GMS region by the end of 2030.

In the phase I of malaria elimination [3], the implementation of entomological surveillance of vector bionomics was developed by the Centre for Malaria, Parasitology and Entomology (CMPE). The CMPE staff selected 13 districts from 8 provinces, 3 from the north and 5 from the southern part of the country as sentinel sites. Each year from 2018 to 2020, entomological surveillance, in collaboration with provincial and districts malaria units, and technical partners (World Health Organization, WHO), was carried out at these sentinel sites. Continuous collections of entomological data from sentinel sites provides information on vector densities, changes in vector behavior and helps to generate an evidence base to inform an effective vector management strategy. Vector surveillance was also carried out in active transmission foci during malaria outbreak response activities, which was mainly in the south. This collection method was devised in order to have vector data that complimented recent epidemiological data, in an effort to try and provide a more complete picture of the transmission dynamics in areas with active and ongoing transmission, and to inform policy on the selection criteria for entomological surveillance sites.

The effectiveness of any vector control intervention is greatly influenced by the ecology and behaviour of malaria vectors [4]. In fact, entomological surveillance is crucial to guide the choice of control strategy to be applied on the ground. It is highly helpful that recent data be available on (i) vector infectivity rate, composition, diversity and abundance, including sibling species and (ii) the spatial and temporal distribution patterns of potential vectors and (iii) presence/absence of any resistance to public health insecticides [5]. The main barriers to continued vector control of malaria are now the lack of routine programmatic entomological monitoring, and capacity for data processing, analysis and interpretation in endemic countries.

The primary vectors in Laos are *Anopheles dirus*, *Anopheles maculatus*, and *Anopheles minimus*, while secondary vectors include *Anopheles aconitus*, *Anopheles barbirostris*, *Anopheles nivipes* and *Anopheles philippinensis* [5]. The latest malaria vector bionomic study in Laos was implemented from 2013 to 2015 in villages in ten provinces during the wet and dry seasons [5]. Key results showed that people living in rural and remote areas are consistently exposed to vectors throughout the year, especially outside. Thus, stressing the need for new tools concerning residual transmission outside in the villages between 18 and 22 h, before people sleep inside with LLINs. In the present study the CMPE also implemented a pilot study on vector bionomics in cultivation sites (i.e. rice fields, cassava and other local crop plantations) near the forest.

In this paper, the vector collection method and the results are presented, including a specific analysis of collection in areas at high risk of malaria parasite transmission (API > 1) in southern villages and in cultivation sites away from the village and adjacent to forested areas. This is the first time that results from collections undertaken as part of an outbreak response, as well as collections from cultivation sites, are reported for Lao PDR. Key recommendations to national authorities to enhance malaria control and accelerate malaria elimination in Laos by using entomological surveillance as a cornerstone are presented. It should be noted that this data was collected, analysed and utilized as part of the national malaria programmes ongoing elimination operations, and not as part of a research project. This means that there are some limitations to the methods and analysis, however it is important that the data and conclusions that this work generated contributes to the global evidence base and the dissemination of programmatic operational practices, as part of the endeavour to eliminate malaria.

## Methods

### Locations

**Fig. 1:**
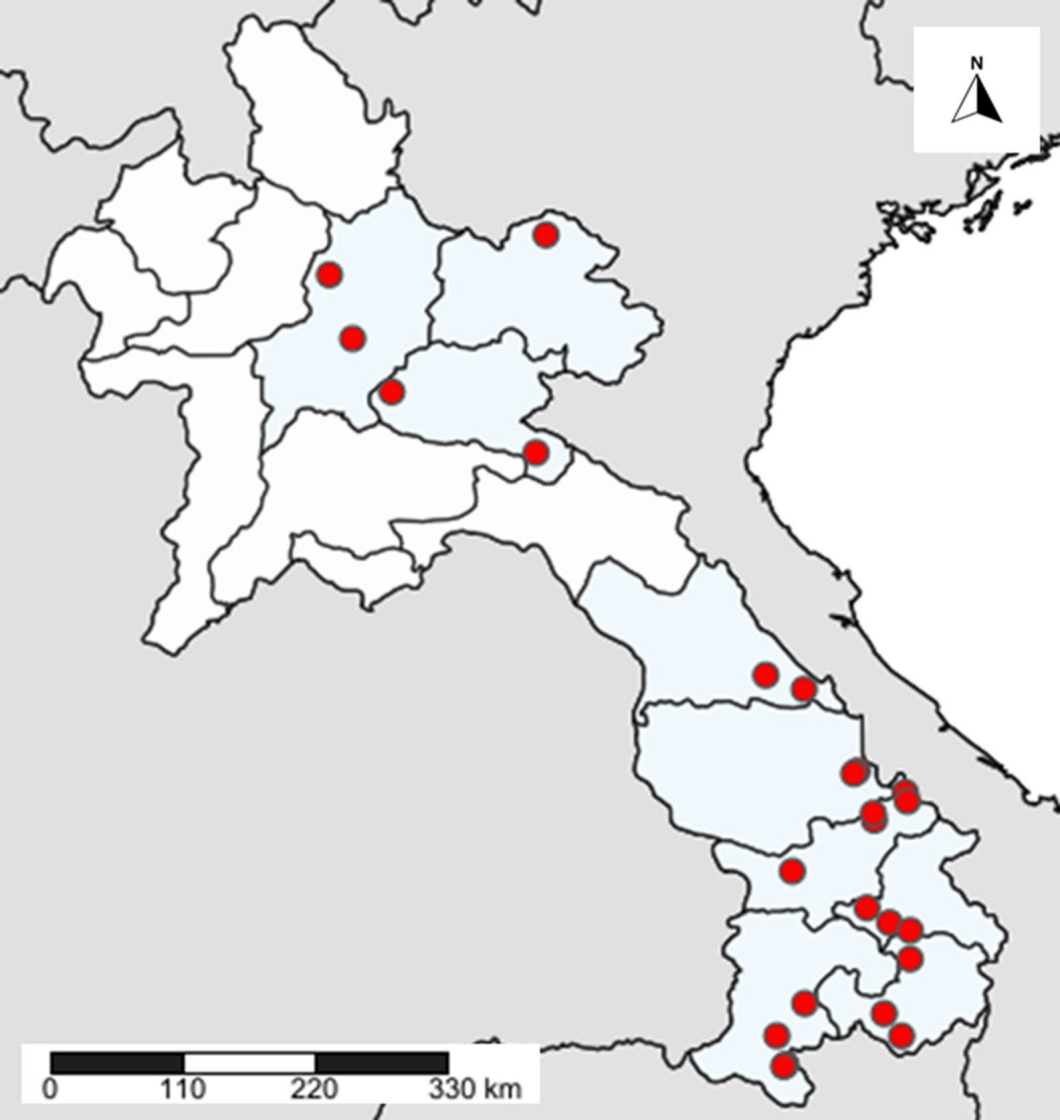
Location of mosquito collection sites during 2018–2020 in Laos. (simplemap^®^)

The CMPE selected sentinel sites from 8 provinces, 3 from the north (Huaphanh, Luang Prabang, and Xiengkhuang) and 5 from the southern part of the country (Attapeu, Champassak, Saravane, Savanakhet, and Sekong). Each location was selected where health facility catchment areas (HFCA) reported high historical burden of malaria. Before 2018, the epidemiological data were reported by these health facility catchment areas and after, the cases were reported by villages. From 2018 to 2020, entomological surveillance, in collaboration with province and district malaria units and technical partners, was carried out at these sentinel sites annually. Vector surveillance was also carried out in active foci (as part of malaria outbreak response or foci response). Spot-checks may be conducted randomly or opportunistically in selected areas or to supplement routine observations. Table 1 and Fig. 1 show the locations selected for sentinel sites and malaria response. Collections were implemented in 24 different villages or cultivation sites. All the villages are located in rural forested areas with different ecotypes characteristics depending on the geographic locations in the country (Fig. 1). With a total area of 236,800 square kilometres, the country is divided into three distinct regions: diverse mountains, plateaus and plains along the Mekong region. Around three-quarters of Laos comprises mountains and plateaus, especially in the areas of the North and South-East. Northern Laos is dominated by rough mountains, jungles and agricultural areas. The plain region is located along the Mekong River and forms the other quarter of the country [6].

**Table 1:** Mosquito collection sites in Laos (2018–2020)

Province	District	Village	Latitude	Longitude	Selection
Champasack	Khong	Nafang	14.46467604	105.86329615800027	Malaria response
	Khong	Navaeng	14.23896916	105.93322870917459	Malaria response
	Khong	Nasaenphan	14.22401106	105.9173030254233	Spotcheck
	Pathumpone	Phakkha	14.71931192	106.08290470983852	Sentinel site
Attapeu	Phouvong	Lamong	14.46282674	106.84352244770805	Malaria response
	Sanxay	Moun	15.06997154	106.91063591735988	Sentinel site
	Phouvong	Vonglakhon	14.63957947	106.70664226259188	Sentinel site
Sekong	Lamam	Navasaen	15.35482896	106.74915242081656	Malaria response
	Lamam	Kasangkang	15.29365664	106.91154867500016	Malaria response
	Tateng	Gnokthong	15.47243938	106.57251145848761	Sentinel site
Saravane	Ta oi	Toumlithong	16.16287252	106.62886723005104	Malaria response
	Samuay	Phinxe	16.38026876	106.87216235905605	Malaria response
	Samuay	Lava-nua	16.31165203	106.88910438975425	Sentinel site
	Vapee	Napho	15.76063237	105.98481030353292	Sentinel site
Savannakhet	Nong	Houp	16.55009712	106.49588152607366	Malaria response
	Nong	Tamlong	16.52989962	106.4677218282346	Malaria response
	Nong	Pane	16.21637962	106.62113804826237	Sentinel site
Khammuane	Bualapha	Thangbaeng	17.19405963	106.07799109053641	Malaria response
	Bualapha	Napoung	17.30432363	105.77616967384732	Spotcheck
Xiengkhuang	Mork	Namyiam	19.05592613	103.97084923668169	Sentinel site
	Phoukoud	Chomsi	19.53496715	102.83719467221466	Sentinel site
Luangprabang	Nambak	Namkha	20.45733256	102.34792903189438	Sentinel site
	Phonxay	Thakham	19.95481194	102.52987688595547	Sentinel site
Huaphanh	Ett	Kang	20.77145923	104.04903036486623	Sentinel site

### Mosquito collections

Routine entomological monitoring at sentinel and high transmission sites was initiated in July 2018. The techniques used for the mosquito collection were indoor and outdoor human landing collections (houses and cultivated areas) and cattle baited net trap collections, following the National Vector Surveillance guideline. The cultivation sites (CS) were selected as part of a malaria outbreak response (Table 1). These areas were outside the villages where recent malaria patients had spent the night.

### Human landing collection (HLC; house: indoor and outdoor; cultivation sites)

Villages were divided into three zones from a central axis to select at random one house per quadrant. The three houses were located at least 30 m from each other. The mosquito collections were implemented for 12 h from 18:00 to 06:00 for three consecutive nights inside and outside the houses. A rotation of collectors between homes was carried out and coordinated by the supervisors. Two shifts rotated, one from 6 pm to midnight and the other from midnight to 6am. Using glass tubes, the collectors captured mosquitoes from their exposed legs. All the mosquitoes collected throughout the night were separated hourly and kept in glass tubes for identification. The number of mosquitoes caught hourly was recorded by supervisors.

Collections done as part of an outbreak response were generally done within the village. However, collection at the cultivation sites were outside the village, preferably at the place where the malaria patient identified stayed overnight during the 28 day period before they presented with symptoms. The same time and methodology of collection was used as the village collections.

Additional file 1: Table S1 shows the number of night collections in the house, at the cultivation sites (CS) and on cattle (CBC) between 2018 and 2020. There were more collections during the rainy season (N = 16) compared to the dry season (N = 5). Only three collections were implemented in 2019 because of a lack of funding for entomological surveillance operations. In total, the number of collection days was 25 for HLC in the cultivation sites on humans, 65 on CBC and 70 for HLC in the houses.

### Cattle baited net trap collection

Coordination was done with the village authorities to lend livestock for the collection. After selection of an appropriate area (outside of the HLC areas), cattle bait collections were carried out by placing a 24 m long net around the animal. The animal was put into the trap before the sunset. Adult mosquitoes landing on the net were collected by using aspirators and flashlights by a collector for 15 min each hour between 1800 and 0600.

### Morphological mosquito identification

The morning following the collections, mosquitoes from both HLC and Cattle Bait Collection (CBC) were morphologically identified to genus (*Aedes*, *Anopheles*, *Armigeres* and *Culex*) and to species or group/complex in a field laboratory, using microscopes and appropriate identification keys for Southeast Asian anophelines [7]. Identification was carried out on site by qualified entomologists from the CMPE.

### Calculations of malaria mosquito bionomics

The human biting rates (HBR) and the cattle biting rates (CBR) as well as anthropophagic and endophagic indexes (AI and EI, respectively) were calculated according to the following formulas:

HBR = No. mosquitoes collected on human volunteers/No. of human-nights; CBR = No. mosquitoes collected on cow bait/No. of cow-nights; HBR indoors = No. mosquitoes collected on human volunteers indoors/No. of human-nights indoors; HBR outdoors = No. mosquitoes collected on human volunteer outdoors/No. of human-nights outdoors; Anthropophagic Index (AI) = HBR/(HBR + CBR); Endophagic index (EnI) = HBR indoors/(HBR indoors + HBR outdoors); Exophagic index (ExI) = HBR outdoors/(HBR indoors + HBR outdoors).

### Epidemiology

Epidemiological data were extracted from the national malaria database: District Health Information System, Version 2 (DHIS2). Population data were taken from the data collected as part of the LLIN distribution campaign in 2019 and the annual population growth rate of 1.4% [8] was applied to other years. Epidemiological and population data for the health facility catchment area were used rather than the village level data, due to the limited availability of quality village level epidemiological data prior to 2018, as well as the lack of reliable village level population data at any time period. Annual parasite incidence (API) was calculated as the number of cases reported in that year dived by the population multiplied by 1000. Annualized monthly API was calculated as the number of cases reported in that month, dived by the population divided by 12, and multiplied by 1000. Annual API and annualized monthly API were calculated by health facility catchment area of the entomological surveillance collection site. Annual API was used to assess the historical trend in malaria burden (which influenced the selection of the surveillance site) and annualized monthly API were used to assess the malaria transmission in the three months prior to, and post vector surveillance.

Annualized monthly API were plotted for malaria response sites and sentinel sites separately. Three-month average API were calculated by summing the cases reported in the collection month and the month before and after, dividing by the annual population dived by four, and multiplying by 1000. The three-month average API was plotted against total HBR for malaria response sites and sentinel sites separately.

## Results

### Anopheles diversity, abundance and distribution

A total of 5601 adult mosquitoes representing 25 different *Anopheles* species were collected and morphologically identified (Table 2). The primary vectors *Anopheles dirus *sensu lato (s.l.) (Leucosphyrus group)*, An. maculatus *s.l. (Maculatus group) and *An. minimus *s.l. (Funestus group) constituted 3.32% (n = 186), 14.75% (n = 826) and 7.27% (n = 407) of all *Anophele*s spp. collected, respectively. Adults of *An. maculatus *s.l. and *An. minimus *s.l. were found in all provinces. The other primary vector, *An. dirus* was found in all the provinces except Huaphanh, Luang Prabang and Xiengkhuang, the northern provinces of Laos. The most abundant secondary malaria vector species was *An. barbirostris* which constituted 27.9% of all *Anopheles*. The other secondary vectors, *An. aconitus*, *An. nivipes* and *An. philippinensis*, represented 1.3%, 19.03% and 4.78%, respectively. *Anopheles hyrcanus*, the most abundant non-malaria vector, represented more than 8% of the total *Anopheles* collected. The highest number of *Anopheles* spp. collected was in Sekong province, in the south part of Laos, with 1909 mosquito specimens, representing 34.08% of the total *Anopheles* collected. Additional file 1: Table S2 shows the details of the collection per species for each village and Additional file 1: Table S3 shows the collections in the cultivated sites.

**Table 2:** Species diversity and abundance of morphologically identified *Anopheles* sp. mosquitoes collected in Laos (2018–2020)

Vector Status	Species	Attapeu	Champasack	Huaphanh	Khammuane	Luangprabang	Saravane	Savannakhet	Sekong	Xiengkhuang	Total	*Percentage*
Primary	*An. maculatus *s.l.	19	25	4	17	34	222	92	409	4	826	*14.75*
	*An. minimus *s.l.	4	10	2	105	0	152	21	56	57	407	*7.27*
	*An. dirus *s.l.	3	6	0	14	0	27	9	127	0	186	*3.32*
Secondary	*An. barbirostris *s.l.	56	61	24	14	258	25	4	384	739	1565	*27.94*
	*An. nivipes *s.l.	85	56	37	12	114	127	38	577	20	1066	*19.03*
	*An. philippinensis*	4	2	0	3	110	52	6	89	2	268	*4.78*
	*An. aconitus*	3	7	2	4	0	15	8	28	6	73	*1.30*
Non vector	*An. hyrcanus *s.l.	18	5	112	3	140	165	0	43	0	486	*8.68*
	*An. kochi*	3	3	101	2	0	129	1	159	43	441	*7.87*
	*An. vagus*	1	15	147	0	0	2	0	3	0	168	*3.00*
	*An. tessellatus*	0	58	0	4	0	1	1	24	0	88	*1.57*
	*An. jeyporiensis*	1	0	0	6	0	0	0	10	0	17	*0.30*
	*An. umbrosus*	2	0	0	0	0	3	1	0	0	6	*0.11*
	*An. argyropus*	0	0	2	0	0	0	1	0	0	3	*0.05*
	*An. culicifacies*	0	0	0	0	0	1	0	0	0	1	*0.02*
	Total	199	248	431	184	656	921	182	1909	871	5601	*100*
	*Percentage*	*3.55*	*4.43*	*7.70*	*3.29*	*11.71*	*16.44*	*3.25*	*34.08*	*15.55*	*100*	

**Fig. 2:**
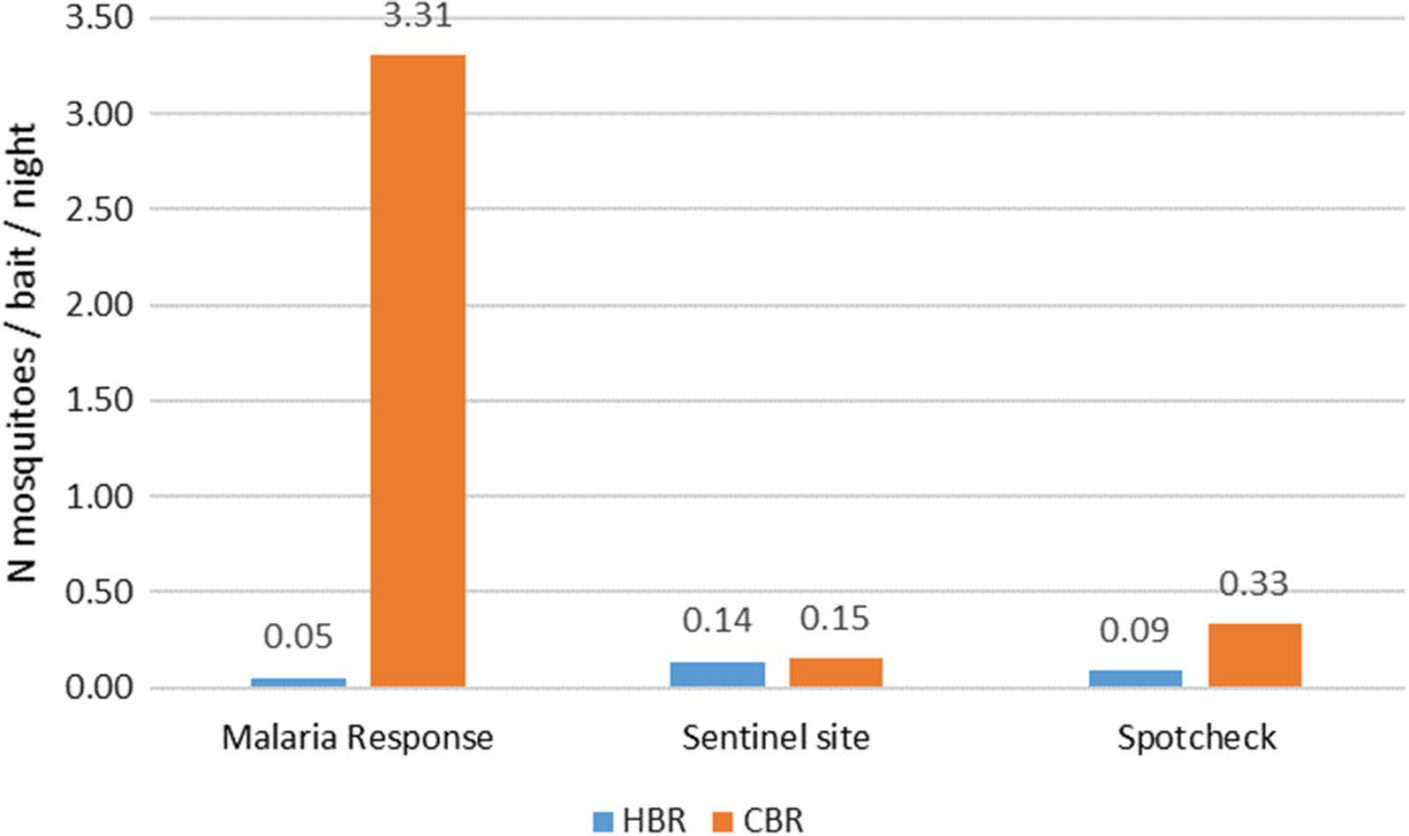
Human and CT biting rates in the malaria response sites, sentinel sites and spotcheck sites in the southern provinces of Laos (primary and secondary vectors)

Figure 2 shows the human and cattle biting rates by surveillance type in the southern provinces where malaria transmission is the highest (Attapeu, Champasack, Khammuane, Saravane, Savannakhet and Sekong). In the malaria response locations, the CBR were largely higher than the HBR (3.31 > 0.05 N mosquitoes/bait/night). In the sentinel sites the biting rates were similar (BR = 0.15) and the CBR were 3 times higher than the HBR.

Additional file 1: Fig. S1 in illustrates the abundance of primary and secondary vectors in the provinces sampled. Primary vectors were the most abundant in Sekong (Average number = 197 Anopheles captured per collection over 2018–2020) followed by Saravane (avg. = 134). The lowest abundance was observed in the northern provinces (Xiengkhuang, Luang Prabang, and Huaphanh; 1 collection per province). The highest abundance of secondary vectors was recorded in Sekong (avg. = 359) and in Luang Prabang (avg. = 482), followed by Saravane (avg. = 219).

Additional File 1: Fig. S2 shows that less *Anopheles* adults (primary and secondary vectors) were captured during the dry season (avg = ; 164 specimens per collection) compared to the rainy season (avg. = 223 per collection). It should be noted that more collections were conducted in the rainy season (N = 16) compared to the dry season (N = 5) and a very large amount was collected in Sekong, Saravane and Luang Prabang during the rainy season. The highest abundance of primary and secondary vector was measured in Sekong during the rainy season (N = 1670, 2 collections).

### Host preference and behaviour

#### Host preference

Table 3 shows the abundance of *Anopheles* mosquitoes according to which host they were collected on. There was a high diversity of *Anopheles* species (n = 15) and the most abundant species were primary and secondary malaria vectors. About 78.5% of *Anopheles* (N = 4399) were collected on cattle, whereas 21.5% were on humans (N = 1202; 4.7% in the cultivation sites). Among the primary vectors, *An. maculatus* was the most abundant species collected on both cattle and humans (N = 826). In the cultivation sites, *An. dirus* was the most abundant species (N = 96), followed by the other primary vectors *An. minimus* (N = 64), and *An. maculatus* (N = 44). *An. barbirostris*, *An. nivipes* and *An. hyrcanus* were the most abundant species collected at the houses with 278, 159 and140 specimen, respectively.

**Table 3:** *Anopheles* species collected and morphologically identified in Laos based on bait and location (N and %) (CS: cultivation site)

Anopheles species	CT	HLC CS	HLC house	Total	*Percentage*
*An. barbirostris *s.l.**	1256	31	278	1565	*27.9*
*An. nivipes *s.l.**	900	7	159	1066	*19.0*
*An. maculatus *s.l.*	691	44	91	826	*14.7*
*An. hyrcanus *s.l.	334	12	140	486	*8.7*
*An. kochi*	427	0	14	441	*7.9*
*An. minimus *s.l.*	265	64	78	407	*7.3*
An. philippinensis**	179	8	81	268	*4.8*
*An. dirus *s.l.*	30	96	60	186	*3.3*
*An. vagus*	155	0	13	168	*3.0*
*An. tessellatus*	86	0	2	88	*1.6*
*An. aconitus*	53	0	20	73	*1.3*
*An. jeyporiensis*	13	0	4	17	*0.3*
*An. umbrosus*	6	0	0	6	*0.1*
*An. argyropus*	3	0	0	3	*0.1*
*An. culicifacies*	1	0	0	1	*0.0*
Total	4399	262	940	5601	*100*
*Percentage*	*78.5*	*4.7*	*16.8*	*100*	

*Primary vector, **Secondary vector

**Fig. 3:**
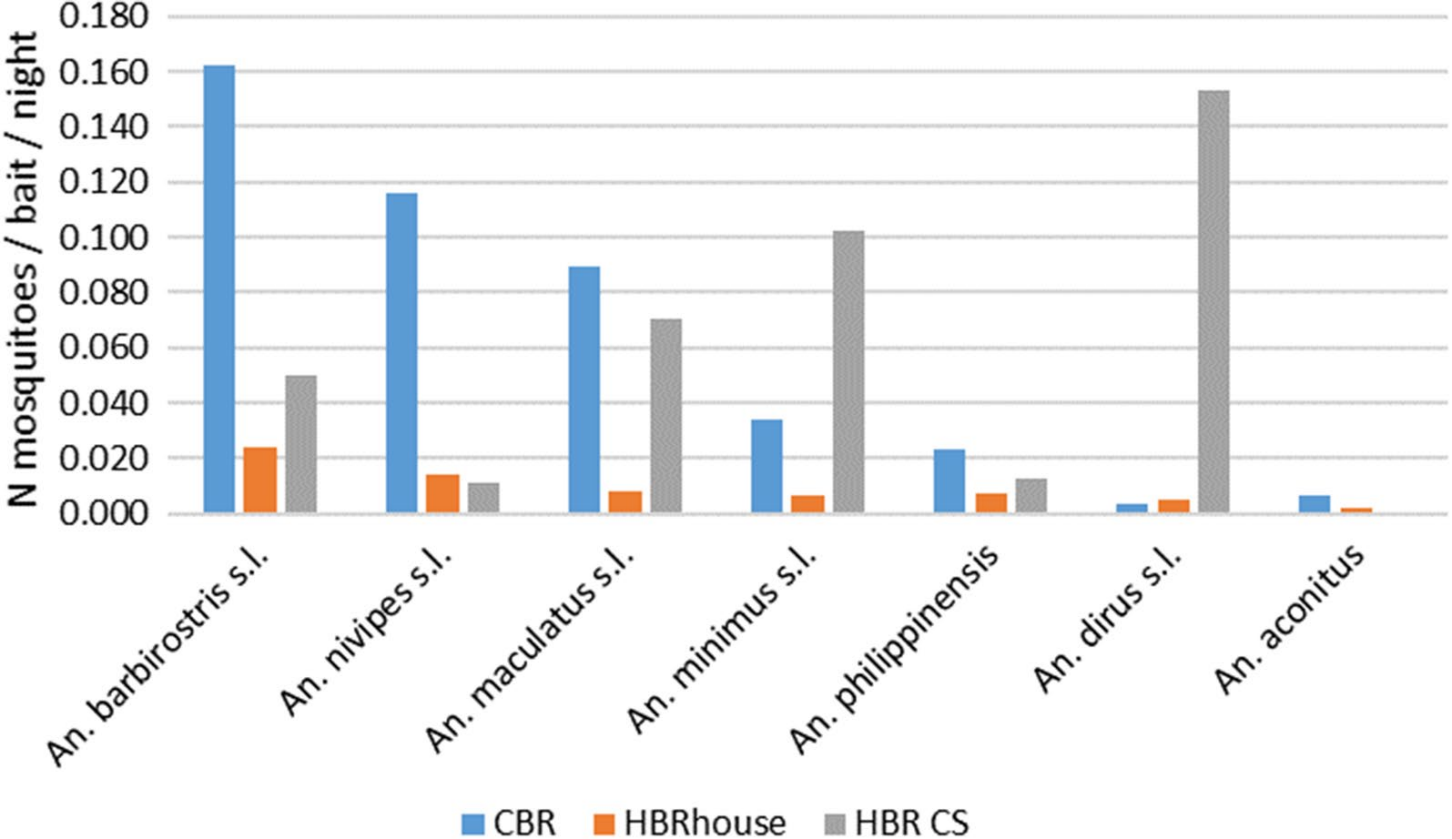
Host biting preference of primary and secondary vectors in Laos (data from 2018 to 2020). Cow biting rates (CBR), Human biting rates (HBR) house and CS

Figure 3 shows the cattle (A), human house (B) and human cultivation site (C) biting rates of primary and secondary vectors. The seven species were collected in all cultivation sites, homes and on CT except *An. aconitus*, which was not found in the cultivation sites. It is clear that *An. dirus* is predominant in the cultivation areas (N = 96, 51.6%) despite the low number of collection days (n = 25) compared to CBC and HLC in the houses (N = 65 and 70 collection days, respectively). Cow biting rates in the villages were much higher (> 0.025) than the human biting rates in the houses in the villages except for *An. dirus* and *An. aconitus* (< 0.007). The HBR in the cultivation sites are similar in range to the CBR with HBR varying from 0.011 for *An. nivipes* to 0.154 for *An. dirus*. The other two primary vector showed the highest HBR in the cultivation sites with HBR = 0.102 and HBR = 0.070 for *An. minimus* and *An. maculatus*, respectively. The highest CBRs were reported for *An. barbirostris*, *An. nivipes *s.l. and, *An. maculatus *s.l. (0.162 > CBR > 0.089).

**Fig. 4:**
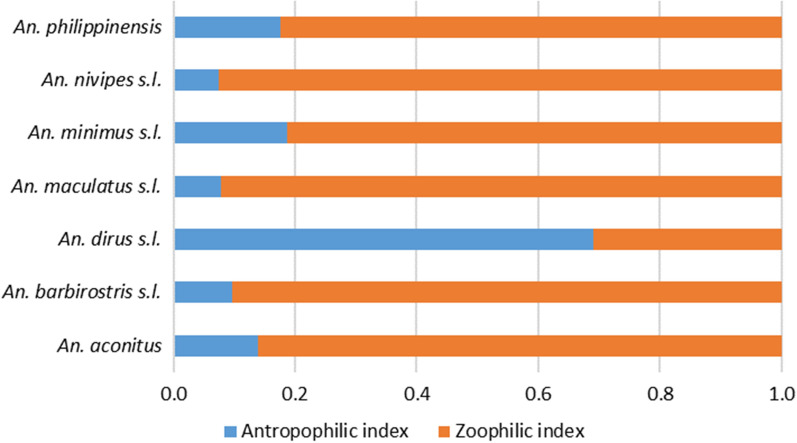
Zoophilic and Anthropophilic index of the *Anopheles* spp. collected in Laos. Zoophilic index calculated as CBR/(CBR + HBR)

*Anopheles dirus* was the most anthropophilic mosquito species collected with an anthropophilic index (AI) of 0.69 compared to a zoophilic index (ZI) of 0.31 (Fig. 4). The two other primary vectors, *An. maculatus *s.l. and *An. minimus *s.l. showed higher zoophilic indices (ZI = 0.92 and 0.81, respectively). All secondary vectors were also strongly zoophilic (> 0.82%).

#### Behaviour

Additional file 1: Table S4 shows the biting times and numbers of all the *Anopheles* species collected between 6 pm and 6 am indoors, and Additional file 1: Table S5 shows the biting times of mosquitoes collected outdoors. A total of 940 specimens were captured. The number varied from 0 to 159 specimens collected all night long. The peak activity of all *Anopheles* species included was between 20 and 21 h and was similar for only primary and secondary vectors. The results show that vectors were active through the entire night.

**Fig. 5:**
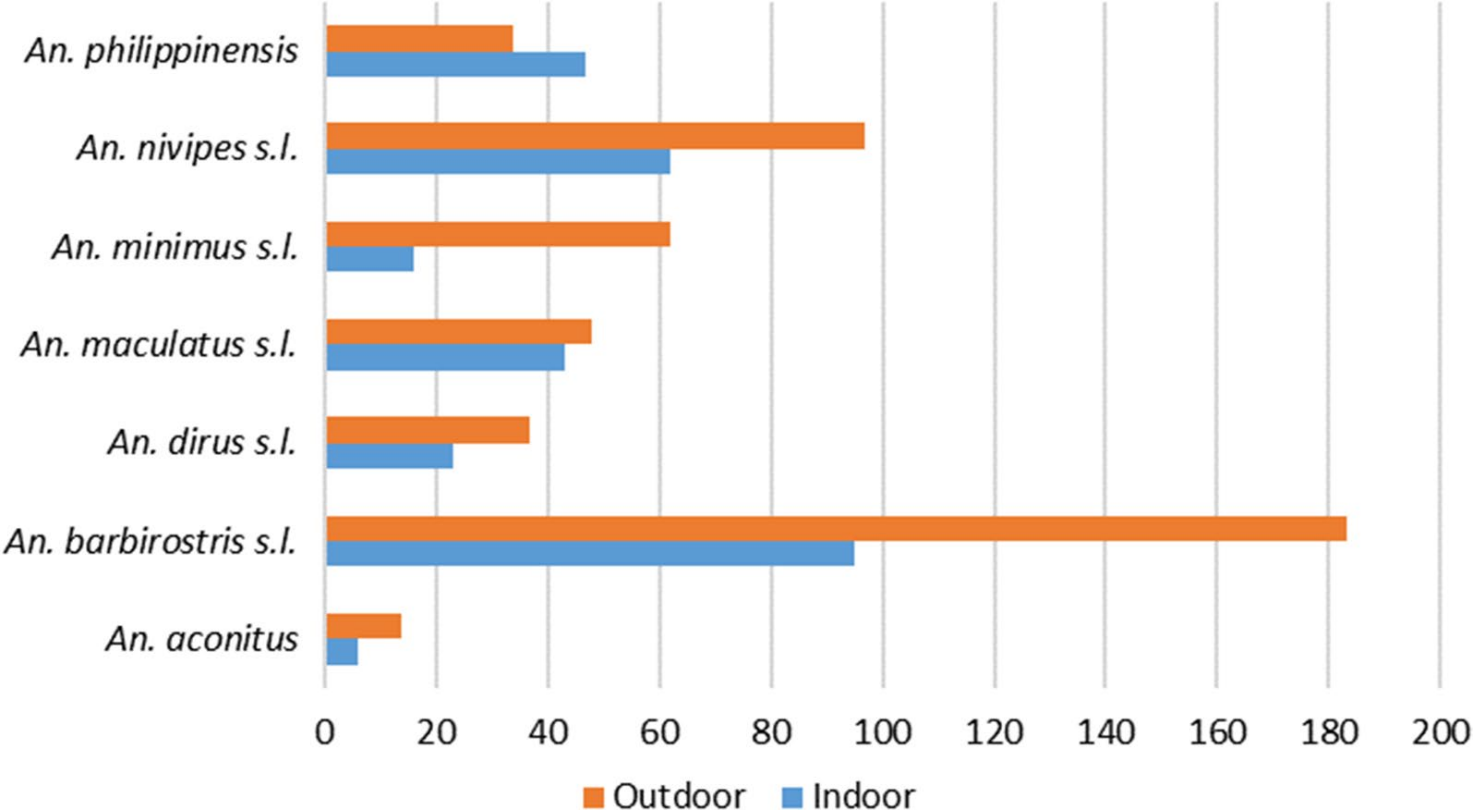
Abundance of *Anopheles* spp. mosquitoes (primary and secondary vectors) in Laos indoor and outdoor (2018–2020 data)

Figure 5 shows the abundance of primary and secondary vectors collected indoors and outdoors. Secondary vectors were more abundant (N = 229) throughout the night compared to the primary vectors (N = 538). The most abundant species found indoors or outdoors was *An. barbirostris* (N = 183). *Anopheles aconitus* was the less abundant species both indoors and outdoors (N = 6 and 14, respectively).

**Fig. 6:**
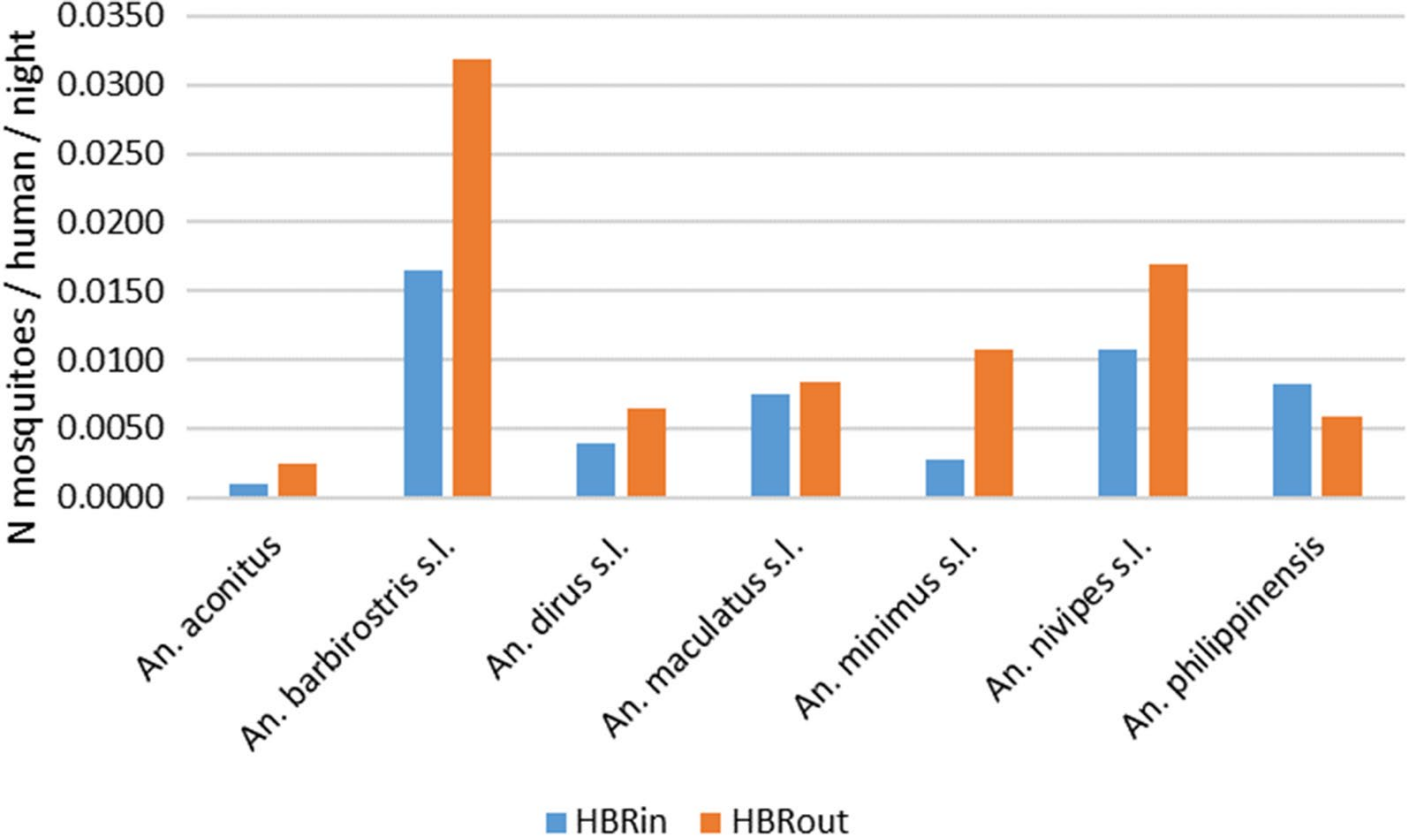
Indoor and Outdoor biting rates of the *Anopheles* mosquitoes collected

The human biting rates indoors (HBRs) varied from 0.001 to 0.0108 (N mosquitoes/human/night) and the HBRs outdoors from 0.0024 to 0.0319 (Fig. 6). The HBRs of *An. barbirostris *s.l. were the highest both indoors and outdoors (HBR = 0.017 and 0.032, respectively) followed by *An. nivipes* s.l. (0.011 and 0.017, respectively). On the other hand, *An. aconitus* had the lowest HBRs for both indoors and outdoors. The indoor and outdoor HBR of *An. maculatus *s.l. was 0.0075 and 0.0084, respectively. The HBRout was higher than the HBRin for *An. minimus* with 0.011 and 0.003, respectively. Overall, the HBRout was higher than the HBRin with values of 0.083 and 0.051, respectively.

**Fig. 7:**
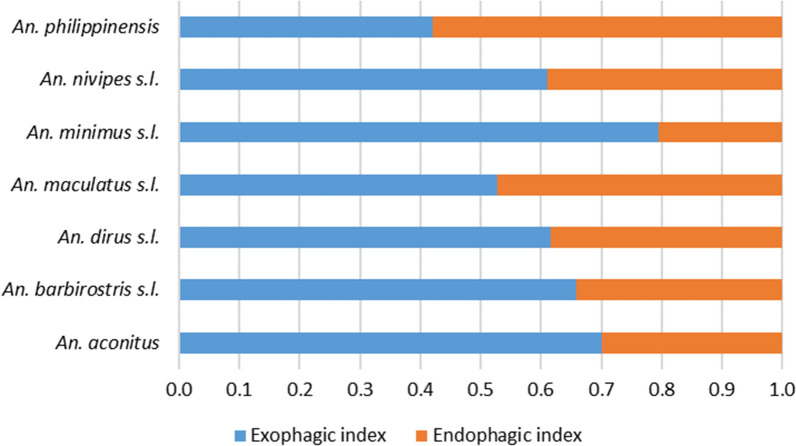
Exophagic index of the *Anopheles* spp. collected in Laos. Exophagic index calculated as HBRout/(HBRout + HBRin)

All species were mostly exophagic (EI > 0.5, Fig. 7). The least exophagic species (i.e. more abundant in indoors collections) were the secondary vectors *An. philippinensis* and the primary vector *An. maculatus* with EIs of 0.42 and 0.53, respectively. The primary vector *An. minimus *s.l. was more active outdoors with EI of 0.79. These results show that people can be constantly exposed to both primary and secondary vectors both inside and outside of their houses throughout the year.

**Fig. 8:**
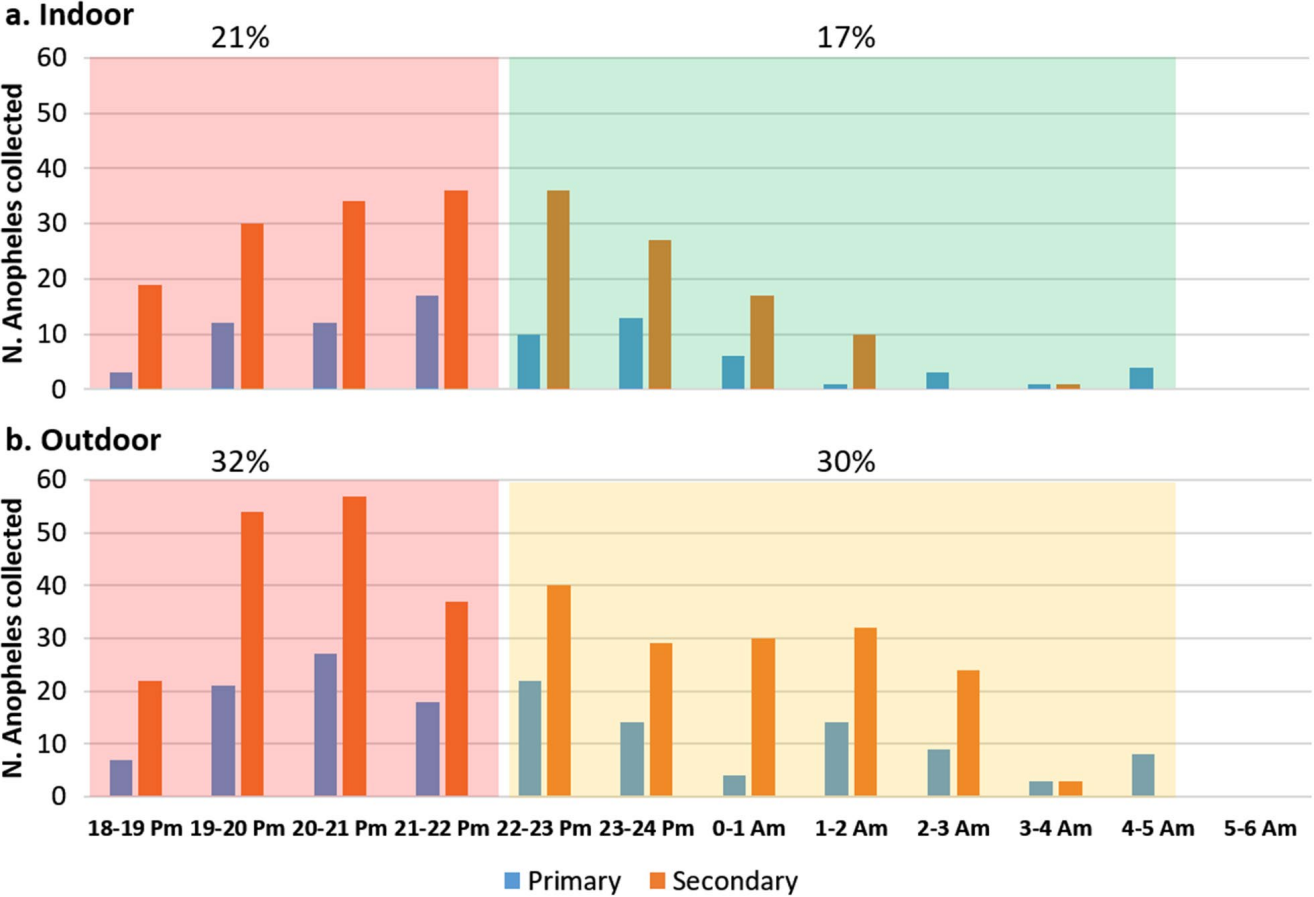
Biting times on human indoor (**a**) and Outdoor (**b**) of the malaria vectors collected between 6:00 PM and 6:00 AM in Laos. In green, time when people are protected by LLINs and in red and orange, periods when people are outdoor without protection in the villages

Result of the biting time indoors of primary and secondary vectors are presented in Fig. 8A. For both primary and secondary vectors, the activity remained high until midnight but was continued until 5am. The peak of biting activity for both type of vectors was between 20 and 21 pm. For outdoors data (Fig. 8B), the results are similar except that the biting activity remains high until 3 am. Primary and secondary vectors were active indoors and outdoors throughout the night. More specifically, 16.8% of the malaria vectors (both primary and secondary) were collected indoors between 10:00 pm and 5:00 am when the people were supposedly sleeping inside under a bed net. Thirty-two percent of primary and secondary vectors were collected outdoors between 6:00 pm and 10:00 pm, when people are still active outside. Figs. S3 and S4 show the detailed biting times of the primary and secondary vectors per species indoors and outdoors, respectively.

**Fig. 9:**
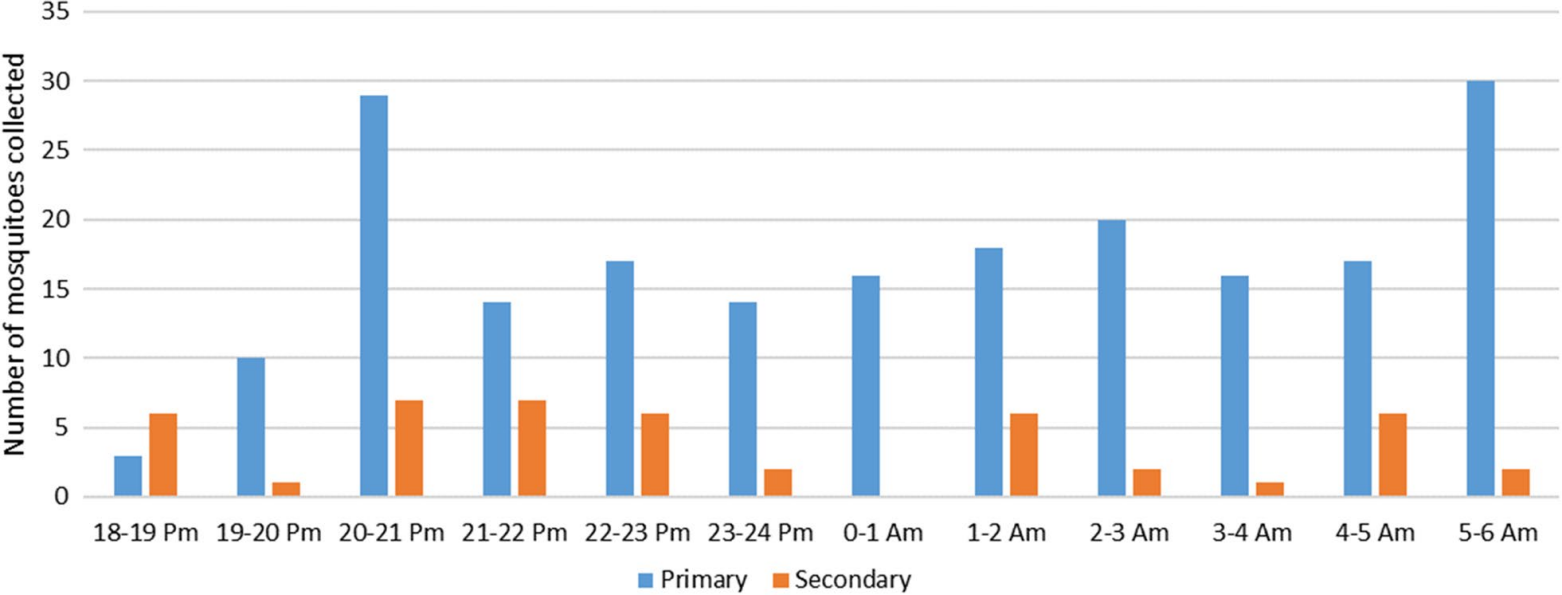
Biting times on human in the cultivation sites of the malaria vectors collected between 6:00 PM and 6:00 AM in Laos

In the cultivated sites, primary vectors were more abundant than the secondary vectors (Fig. 9). Both vector categories were active from 6 pm to 6 am with two peaks of activity between 8–9 pm and 5–6 am for the primary vectors. Fig. S5 shows the biting times per species in the cultivated sites.

**Fig. 10:**
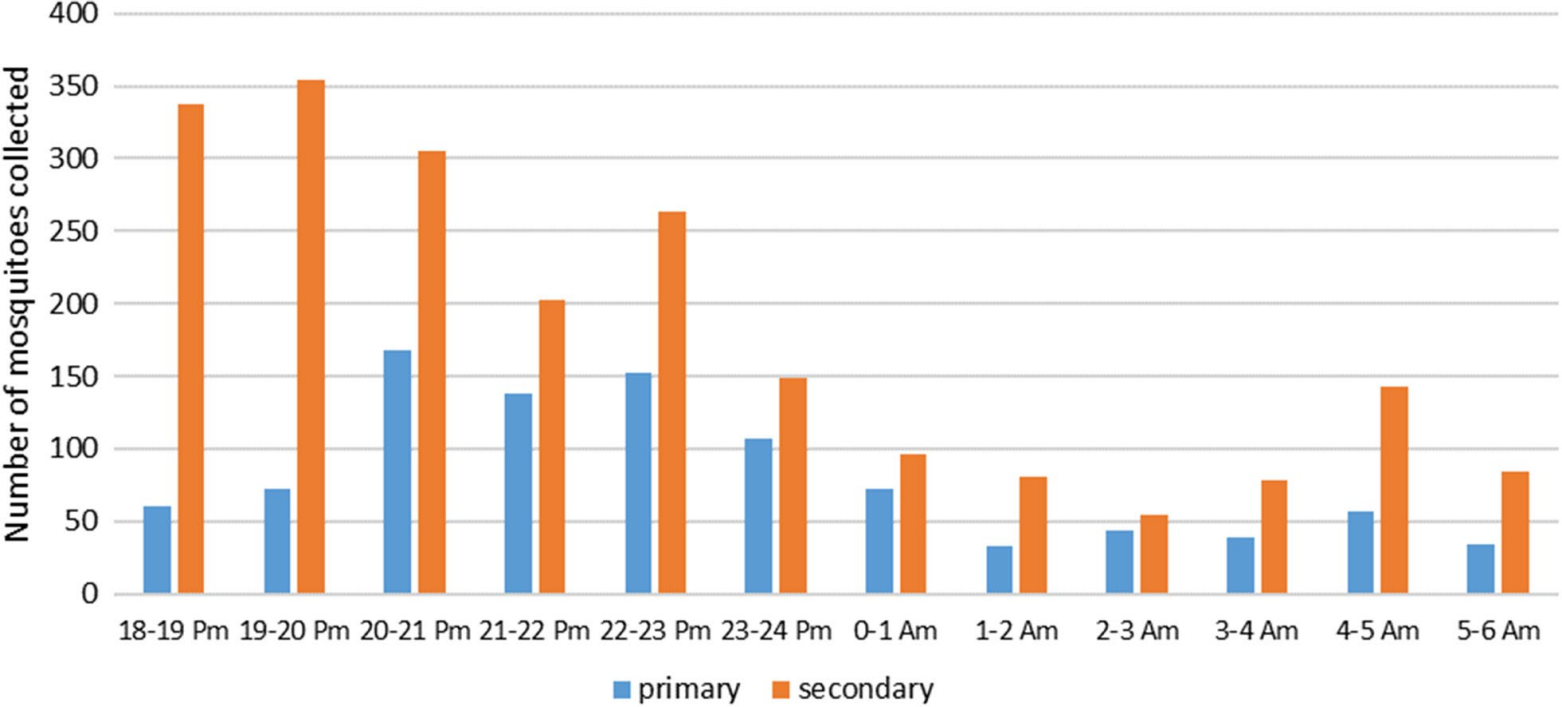
Biting times on cattle of the malaria vectors collected between 6:00 PM and 6:00 AM in Laos

Figure 10 shows the number of *Anopheles* spp. collected on CT bait between 6 pm and 6 am. Primary and secondary vectors were active all night. There were more mosquitoes collected between 6 pm and midnight compared to the rest of the night, but it should be noted that some captures were only implemented between 6 pm and midnight.

#### Epidemiology data results

**Fig. 11:**
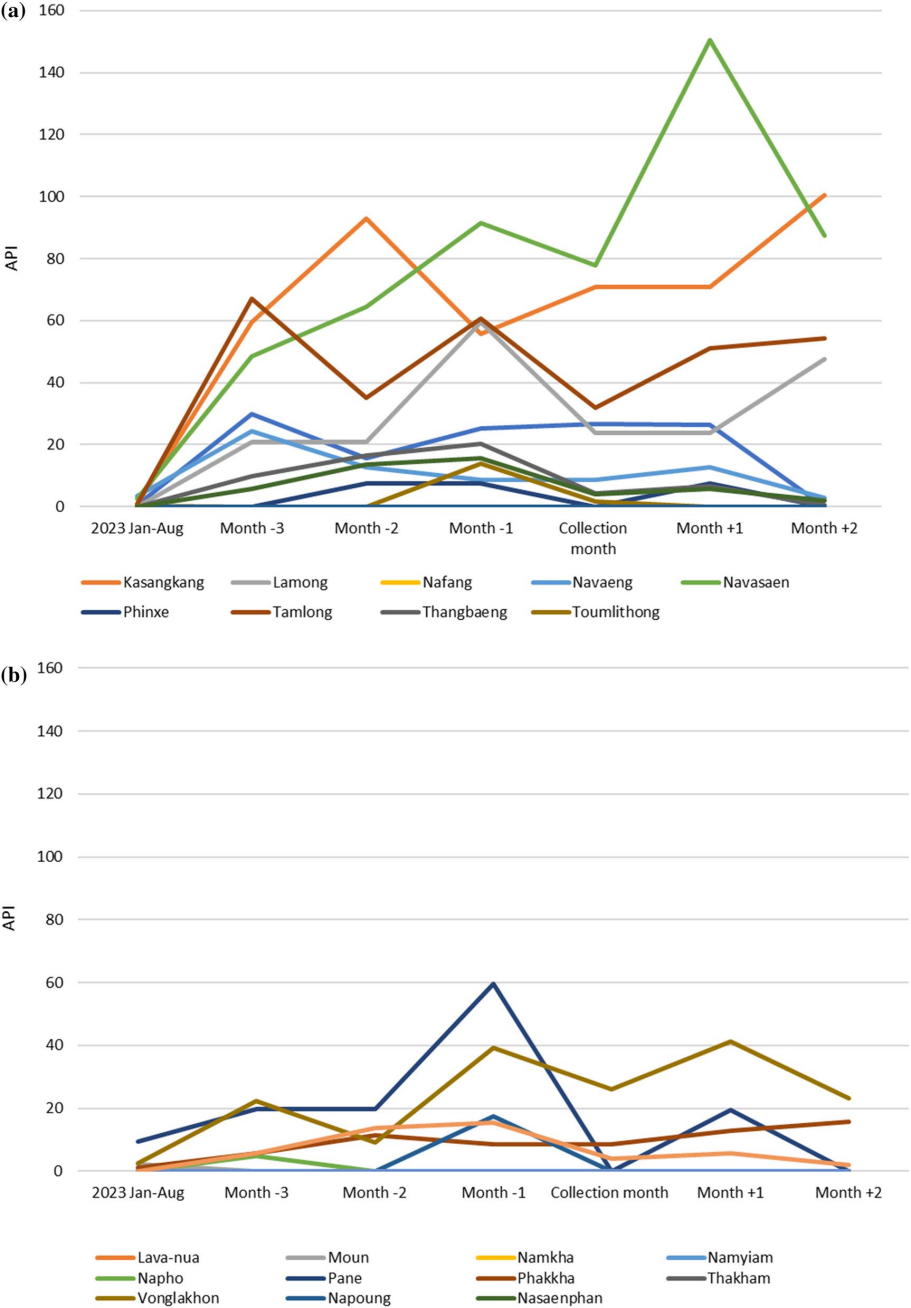
Annual parasite index (API) at the time of survey, three months prior and three months post, in the health facility catchment area: **A** of the malaria response sites; **B** of the sentinel sites

**Fig. 12:**
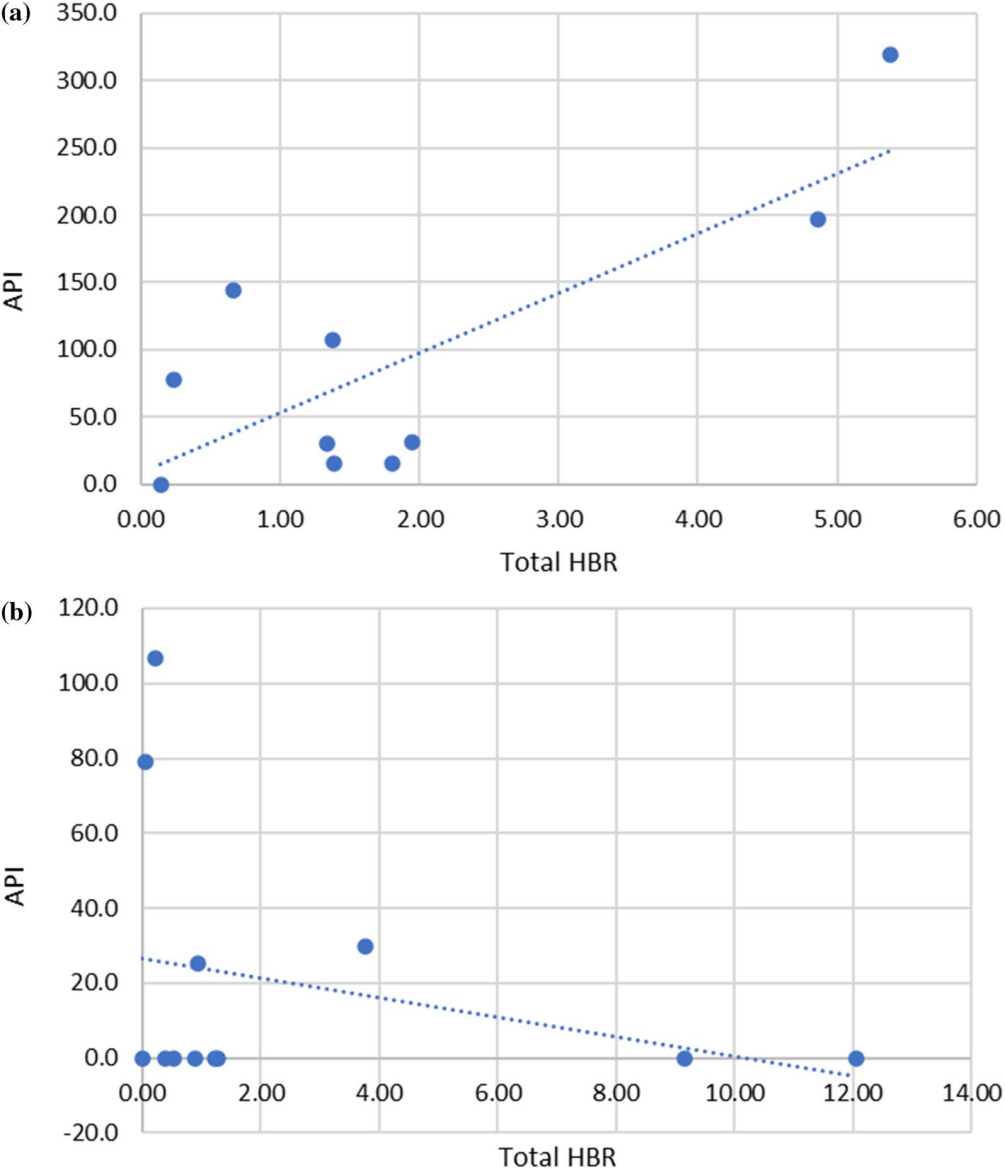
Scatter plot, with line of best fit, for total human biting rate (HBR) compared to annual parasite index (API): **A** at malaria response sites; **B** at sentinel sites

Epidemiological data shown in Table S6 and Fig. 11A and B, demonstrate that malaria transmission in the period around the time of the entomological survey varied by site selection method: 100% (2/2) of spot check sites reported cases in the three months prior or post survey, 90% (9/10) of malaria response sites reported cases with only 46% (5/12) sentinel sites reporting cases. The scatter plot in Fig. 12A and B indicate a possible correlation between API and total HBR in malaria response sites, but not at sentinel sites.

## Discussion

The Lao PDR National Strategic Plan (NSP) for malaria control and elimination for year 2021–2025 emphasizes the importance of entomological surveillance being conducted in areas with high transmission and in active malaria foci in elimination targeted areas. Entomological data that is closely linked to recent epidemiological data is crucial for improving impact, as it contributes to the evidence package that supports operational and strategic decision-making of national malaria programmes as they try to accelerate in their last mile of elimination.

About 78.5% of *Anopheles* mosquitoes were collected on cattle, whereas 21.5% were on humans (4.7% in the forested cultivation areas). They represented 15 different species or species complexes that were already described in Laos [9–13]. The primary vectors *An. dirus *s.l., *An. maculatus *s.l. and *An. minimus *s.l. constituted 3%, 15% and 7% of all *Anophele*s spp. collected, respectively. However, as these were identified by morphological characteristics, they do not reflect the actual primary vector status as not all sibling species may be vectors [5]. Among the primary vectors, *An. maculatus* was the most abundant species collected on both cattle and humans. The abundance of secondary vectors represented more than 52% of all vectors. All these species are mostly zoophilic but can also bite humans. *Anopheles philippinensis* and *An. nivipes* were shown two decades ago to bite both human and animals and were suspected to be responsible for malaria parasite transmission in paddy field zones in Khammouane province [10, 11, 14]. Indeed, both of these species were previously found positive with *P. falciparum* or *P. vivax* in Laos and in other GMS countries [7, 11, 15]. Clearly more work has to be done to determine the behaviour and ecology of secondary vectors and their role in transmission in Laos.

This is the first time that collections were implemented in cultivated sites (CS) outside the villages near the forest where people regularly travel to and spend anywhere from a few nights to 6 months during the agriculture period, which coincides with the wet season, when malaria vectors and malaria cases are more prevalent [5]. In the cultivation sites, primary vectors were more abundant than the secondary vectors (Fig. 10). This is probably due to the fact most of these small seasonal cultivation settlements (locally known as ‘Katos’) are located at the interface of the forest and the crop sites (cassava, rice fields and other plantations). *Anopheles dirus* was the most abundant species, followed by the other primary vectors *An. minimus* and *An. maculatus.* Both vector categories were active from 6 pm to 6 am with two peaks of activity between 8–9 pm and 5–6 am for the primary vectors. These three primary vector species also had the highest HBR in the CS confirming that these populations are at the highest risk and need to be the focus of targeted malaria services, such as personal protection, outreach and access to testing and treatment. Human behaviour observations should be implemented to determine the period of the day or night when people need protection against mosquito infective bites. Furthermore, the study of Vantaux et al*.* [16] showed that 20% of malaria vectors collected in different ecotypes in Cambodia (villages, plantation, forest and forest near village) were active during the day between 06:00 and 18:00 indicating that further surveillance studies should be implemented during the day in these ecotypes in Laos.

In our study, all the vector species were exophagic (EI > 0.5). This trend is also described in surrounding countries such as Vietnam, Thailand and Cambodia where the primary vectors *An. dirus*, *An. minimus* and *An. maculatus* are also mainly outdoor biters [17–19]. Suwonkerd et al*.* [18] explain that this exophagic tendency of vectors is associated with the persistence of malaria transmission among populations with outdoor activities during night time. However, the results showed that vectors are still biting in significant proportions indoors (Fig. 6). Overall, these results show that people are constantly exposed to both primary and secondary vectors both inside and outside of their houses throughout the year. The results corroborate the earlier study in Laos by Marcombe et al*.* [5]. Primary and secondary vectors were highly zoophilic, but they still bite humans throughout the night with a high peak of activity before midnight, both indoors and outdoors. Overall, 17% of the malaria vectors were collected between 10:00 pm and 5:00 am indoors when the people are sleeping. This confirms the continued need for the use of bed nets during this period of the night. Furthermore, the last insecticide resistance study implemented in Laos between 2012 and 2015 showed that no resistance to pyrethroids (used for LLINs) was found in malaria vectors, indicating that these insecticides are still adequate for malaria vector control [20]. However, insecticide resistance has recently been detected in several *Anopheles* species in Cambodia. This indicates that a new screening of resistance against the insecticides used in LLINS in the malaria vectors of Laos is urgently needed [16].

Thirty-two percent of primary and secondary vectors were collected outdoors at times when people are usually awake and outdoors (before 10:00 pm or after 5:00 am), which shows that people are exposed to potentially infectious mosquitoes and the importance of personal protection at these times. The findings showed that the transmission may occur outdoors in the villages, and outside the villages in cultivation sites. Additional tools to target outdoor biting mosquitoes are urgently needed for efficient vector control, as well as strategies to ensure that populations living in seasonal cultivation sites are able to easily access core essential anti-malaria services, such as rapid diagnostics and treatment. Human behaviour observations (HBO) are also needed at high-risk locations in Laos to know the proportion of human sleeping indoors or outdoors with or without bed net in these areas. For example, Martin et al. [21] correlated the HBO and malaria vectors bionomics in Peru and demonstrated that the exophagic feeding of anopheline vectors when analysed in conjunction with human behaviour, indicates a clear gap in protection even with high LLIN coverage. Also, in this study they showed that indoor residual spraying (IRS) may have limited effect because of the lack of indoor-resting anophelines.

Most of the strategies for vector control in Lao are focused on the human environment (bed net and IRS) but the evidence continues to show that a large proportion of mosquitoes are collected on cattle (78.5%, Fig. 2). New vector control tools should address the dynamics of transmission, as well as the ecology of malaria vectors in local contexts. For example, veterinary approaches such as the use of endectocides by injection in livestock [22] or the use of insecticide-treated mosquito nets fenced around cattle [23], or pyriproxyfen-treated polypropylene sheets and resting boxes for controlling mosquitoes in livestock operations [24] may be interesting strategies to target the zoophilic and exophagic malaria vectors in Lao PDR.

The epidemiological data demonstrates that transmission was generally higher in the malaria response and spot check sites compared to the sentinel sites. This finding is expected, as the malaria response sites were targeted due to the occurrence of a malaria outbreak. The spot check sites were selected due to opportunistic collections during other related malaria work activities of CMPE. However, the sample size for spot check sites was small (n = 2) and cases were only reported from 1 to 2 months of the seven months of epi data, respectively, at the spot check sites, therefore results may be due to chance. The visual positive correlation between the HBR and API may indicate that higher HBR increases API, however strong conclusions linking entomological data and epidemiological data in this paper cannot be drawn due to the limitations of the small dataset as well as the fact that mosquitoes collected were not analysed for the presence of malaria sporozoites. Therefore, it was avoided generating strong conclusions or calculating the correlation coefficient with 95% CI. Using the epidemiological data to demonstrate the utility of the site selection methods has been useful however, and subsequently the policy for entomological surveillance now favours malaria response sites and is phasing out sentinel sites.

Limitations include the lack of quality village level epidemiological data for the years prior to 2018 and lack of quality village level population data, therefore epidemiological analysis was done at the HFCA level for consistency over the years.

As Lao PDR intensifies efforts to eliminate *P. falciparum*, CMPE and WHO have developed ‘accelerator strategies’ to complement core interventions. These strategies were scaled up in 2022 and are applied annually in the highest-burden villages with the aim of reducing the parasite reservoir and interrupting transmission in these critical source locations (nowadays only in the southern provinces). Strategies to try and accelerate malaria elimination in Lao include the distribution of new LLINs to target villages (coinciding with the next mass LLIN distribution), targeted distribution of long-lasting insecticidal hammock nets (LLIHNs) to forest goers, targeted drug administration, and intermittent preventive treatment for forest goers (IPTf), among others. Both mobile and static high-risk populations (HRPs) across Laos including forest goers, seasonal field (or cultivation) goers, ethnic minorities, and forest fringe populations continue to pose challenges to elimination and will be targeted through the accelerator strategies to increase their access and use of malaria services and protection.

## Conclusion

Limited expertise and human resources in general entomological methods may further exacerbate the malaria situation, especially at subnational levels. It is crucial to establish a strong domestic capacity to routinely and consistently implement entomological surveillance to accelerate progress toward malaria elimination in Laos. Entomological data should help to guide the national public health malaria strategy and interventions over time and space. With intensive control activities planned in order to reach the elimination targets, regular surveillance of vectors for changes in prevalence of vector species and their behavioural aspect, and regular monitoring of insecticide resistance should continue to be routine activities.

Strong entomological capacity at provincial, district but also at regional (i.e. neighbouring countries) levels will also be critical in order to help prevent re-establishment of malaria once the country has achieved elimination. Investigations of active foci require clear entomological data on the presence and bionomics of malaria vectors around the foci. This information is very important to better understand the transmission (rhythm and intensity) risks, and to inform the type of vector interventions required and the intensity of the vector control response.

Entomological surveillance helps the programme understand where and how transmission is persisting, monitors vector density and biting trends, and effectiveness of vector control, and therefore should be considered as a cornerstone in the fight against malaria in Laos.

### Supplementary Information

**Additional file 1: Table S1.** Number of night of collections in the villages, at the cultivation sites (CS) and on cattle (CT) between 2018 and 2020. **Table S2**. Number of vectors collected per village per year by HLC at the house. **Table S3**. Number of vectors collected per village per year by HLC in the CS. **Table S4**. Biting time in the villages indoors. **Table S5**. Biting time in the villages outdoors. **Table S6.** Annualized parasite index of the health facility catchment area of surveyed villages, showing the trend in annual parasite index (API) and monthly transmission before and after the survey. **Fig. S1**. Total number of primary and secondary malaria vectors collected in Laos, 2018–2020. **Fig. S2**. Biting times of the primary and secondary vectors indoors. **Fig. S3**. Biting times of the primary and secondary vectors outdoors. **Fig. S4.** Biting times of the primary and secondary vectors in the cultivated sites. **Fig. S5**. Biting times of the primary and secondary vectors in the cultivated sites.

## Data Availability

All the data are available upon request.

## Abbreviations

ACT: Artemisinin-based combination therapy
API: Annual parasite index
LLINs: Long lasting insecticidal nets
MoH: Ministry of Health
CMPE: Centre for Malaria, Parasitology and Entomology
WHO: World Health Organization
CS: Cultivation sites
HLC: Human landing catching
HBR: Human biting rates
CBC: Cattle biting collection
CBR: Cattle biting rates
AI: Anthropophagic Index
EI: Endophagic indexes
HBO: Human behaviour observations
IRS: Indoor residual spraying
LLIHNs: Long-lasting insecticidal hammock nets
IPTf: Intermittent preventive treatment for forest goers
HRPs: High-risk populations
HFCA: Health facility catchment areas
